# Non-Specific Inhibition of Ischemia- and Acidosis-Induced Intracellular Calcium Elevations and Membrane Currents by α-Phenyl-*N*-tert-butylnitrone, Butylated Hydroxytoluene and Trolox

**DOI:** 10.3390/ijms15033596

**Published:** 2014-02-27

**Authors:** Christopher Katnik, Javier Cuevas

**Affiliations:** Department of Molecular Pharmacology and Physiology, University of South Florida, College of Medicine, 12901 Bruce B. Downs Blvd., MDC-9, Tampa, FL 33612, USA; E-Mail: ckatnik@health.usf.edu

**Keywords:** ischemia, acidosis, currents, calcium, trolox, BHT, PBN, neurons

## Abstract

Ischemia, and subsequent acidosis, induces neuronal death following brain injury. Oxidative stress is believed to be a key component of this neuronal degeneration. Acute chemical ischemia (azide in the absence of external glucose) and acidosis (external media buffered to pH 6.0) produce increases in intracellular calcium concentration ([Ca^2+^]*_i_*) and inward membrane currents in cultured rat cortical neurons. Two α-tocopherol analogues, trolox and butylated hydroxytoluene (BHT), and the spin trapping molecule α-Phenyl-*N*-tert-butylnitrone (PBN) were used to determine the role of free radicals in these responses. PBN and BHT inhibited the initial transient increases in [Ca^2+^]*_i_*, produced by ischemia, acidosis and acidic ischemia and increased steady state levels in response to acidosis and the acidic ischemia. BHT and PBN also potentiated the rate at which [Ca^2+^]*_i_* increased after the initial transients during acidic ischemia. Trolox inhibited peak and sustained increases in [Ca^2+^]*_i_* during ischemia. BHT inhibited ischemia induced initial inward currents and trolox inhibited initial inward currents activated by acidosis and acidic ischemia. Given the inconsistent results obtained using these antioxidants, it is unlikely their effects were due to elimination of free radicals. Instead, it appears these compounds have non-specific effects on the ion channels and exchangers responsible for these responses.

## 1. Introduction

Brain ischemia causes deprivation of O_2_ and glucose, and a switch from aerobic to anaerobic glycolysis in neurons. These deficiencies ultimately result in ATP depletion, and inhibition of ATP-dependent proteins required for maintaining cellular ionic homeostasis. The production of lactate via anaerobic glycolysis also results in tissue acidosis. This acidosis is known to activate acid-sensing ion channels (ASIC) in neurons, which results in intracellular Ca^2+^ ([Ca^2+^]*_i_*) overload and cell death [1–3]. In addition, there is enhanced glutamate release, and concomitant activation of glutamatergic receptors, such as NMDA receptors, which further promotes intracellular Ca^2+^ overload [4]. One of the consequences of ischemia-evoked calcium dyshomeostasis is mitochondrial dysfunction and the production of reactive oxygen species (ROS) [5]. This interplay between calcium overload, mitochondrial dysfunction and ROS production is a major cause of ischemic injury and a target for potential stroke therapy [6].

It was recently shown that the antioxidant analog of vitamin E, trolox, can decrease injury in a rat model of ischemic stroke, but the mechanism by which this protection occurs remains unclear [7]. One possibility is that the antioxidant activity of trolox prevents ischemia-induced [Ca^2+^]*_i_* overload and resulting cell death. Trolox has been shown to affect calcium handling in cells [8]. While ROS production may contribute to Ca^2+^ overload, both ASIC1a and NMDA receptors have been shown to be regulated by REDOX reagents such that increased oxidation of the channels suppresses currents [9,10]. Thus, it is unclear how a decrease in ROS, or application of antioxidants in general, will affect ischemia-induced [Ca^2+^]*_i_* dysregulation.

Experiments were carried out with structurally distinct antioxidants to determine how they affect [Ca^2+^]*_i_* elevations produced by activation of ASIC1a (acidosis alone), ischemia or a combination of ischemia and acidosis. For our experiments we used α-Phenyl-*N*-tert-butylnitrone (PBN), a spin-trapping molecule which is an effective superoxide scavenger and prevents lipid peroxidation [11], butylated hydroxytoluene (BHT), a synthetic α-tocopherol analogue capable of scavenging hydrogen peroxide [12], and trolox, a water soluble analogue of α-tocopherol shown to prevent protein oxidation by ROS and an effective scavenger of hydroxyl, peroxyl and alkoxyl free radicals and superoxide [13]. Data shown here suggest that each of these molecules has distinct effects on [Ca^2+^]*_i_* elevations produced by the different conditions, and that their effects appear to be the result of off-target activity rather than antioxidant properties.

## 2. Results and Discussion

Ischemia, acidosis and the combination of the two, acidic ischemia, produce increases in [Ca^2+^]*_i_* and activation of inward whole cell currents in cultured cortical neurons isolated from E18 rats. Fluorometric imaging of the calcium indicator Fura-2 and electrophysiological experiments were carried out to determine if these responses were due in part to production of free radicals. Cells were incubated in one of two α-tocopherol analogues, trolox or butylated hydroxytoluene (BHT), or the spin trapping molecule α-Phenyl-*N*-tert-butylnitrone (PBN) prior to and during 2 min applications of either (1) a glucose-free physiological saline solution (PSS) containing NaN_3_; (2) PSS titrated to pH 6.0; or (3) a glucose-free PSS containing NaN_3_ titrated to pH 6.0.

### 2.1. Initial Increases in [Ca^2+^]*_i_* Induced by Ischemia Are Inhibited by PBN, BHT and Trolox

**Figure 1.**
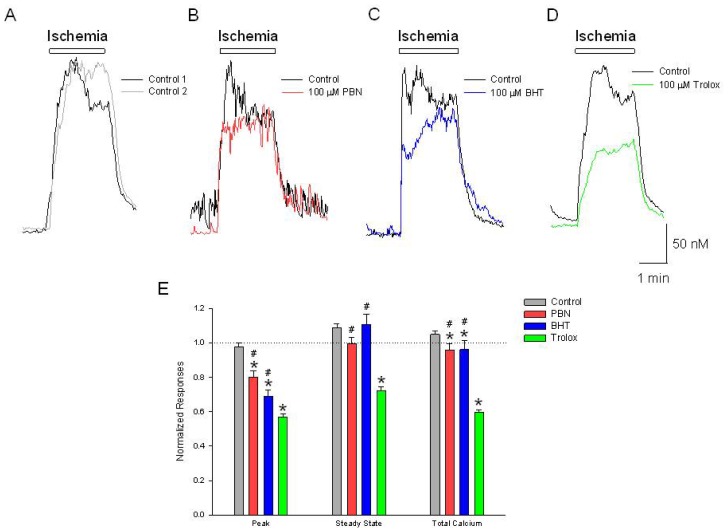
Ischemia produces increases in [Ca^2+^]*_i_*, are differentially inhibited by Phenyl-*N*-tert-butylnitrone (PBN), butylated hydroxytoluene (BHT) and trolox. Representative traces of [Ca^2+^]*_i_*, as a function of time during a (**A**) 2 min application of NaN_3_ in the absence of glucose. The initial ischemia induced elevation in [Ca^2+^]*_i_* (black trace) was reproducible following a 20 min washout period (gray trace); In separate experiments, following the control ischemic responses (black traces), a 20 min incubation in (**B**) 100 μM PBN or (**C**) 100 μM BHT inhibits the initial ischemia-induced increase in [Ca^2+^]*_i_*, (red and blue traces) while (**D**) 100 μM trolox inhibits the initial as well as the steady state increases in [Ca^2+^]*_i_* (green trace); (**E**) Summarizing experiments identical to (**A**–**D**), second responses recorded in the absence and presence of the three antioxidants were normalized to initial control responses and expressed as means ± S.E.M. Peak, initial [Ca^2+^]*_i_* increase relative to baseline; Steady State, [Ca^2+^]*_i_* measured at end of ischemic episode, relative to baseline; Total Calcium, integration of [Ca^2+^]*_i_* increases from beginning of ischemic event to end of record. One way ANOVA comparison was performed on ratios of test responses/control responses. * *p* < 0.5 compared to control, and # *p* < 0.5 compared to trolox. Number of cells measured; Control, *n* = 225; PBN, *n* = 93; BHT, *n* = 131; Trolox, *n* = 107.

In cultured cortical neurons loaded with Fura-2, chemical ischemia produces an immediate increase in [Ca^2+^]*_i_* of 151 ± 5 nM, which decreases to a sustained steady state level of 109 ± 6 nM (Figure 1A). Incubation in 100 μM PBN, BHT or trolox for 20 min inhibited the initial ischemia induced increase by 20%, 31% and 43% respectively (Figure 1B–E). Only trolox, however, decreased the steady state [Ca^2+^]*_i_* obtained at the end of the 2 min azide application, decreasing [Ca^2+^]*_i_* levels by 27% (Figure 1D,E). This initial peak is known to be due to activation of ASIC1a, NMDA receptors and voltage-gated Ca^2+^ channels and is dependent on synaptic transmission, but the mechanisms regulating the sustained component are not well-understood [1,2,14]. The fact that all antioxidants decreased the initial peak suggests that increased ROS production may be regulating this component of the ischemia-induced [Ca^2+^]*_i_* overload, but that the sustained response is not affected by ROS levels. Moreover, trolox may be having an off-target effect to depress this long-lived [Ca^2+^]*_i_* response.

### 2.2. Increases in [Ca^2+^]*_i_* Induced by Acidosis Are Inhibited by PBN and BHT but not Trolox

**Figure 2.**
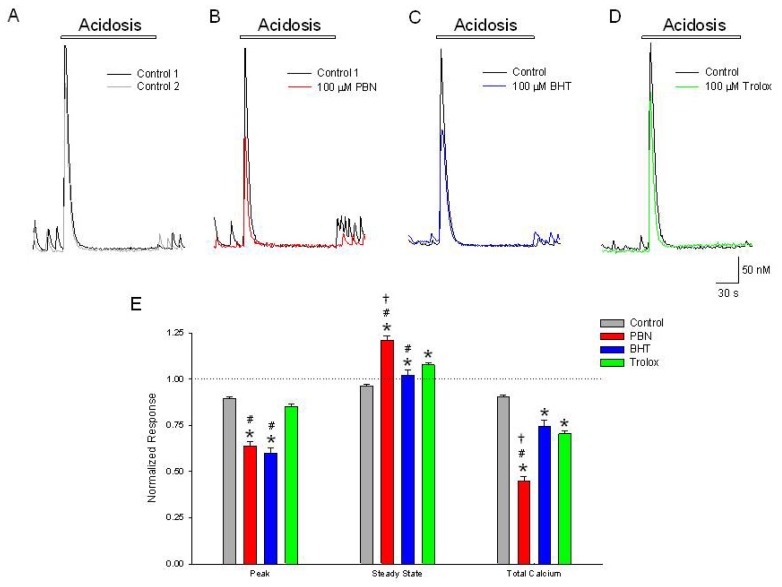
Initial [Ca^2+^]*_i_*, increases in response to acidosis are inhibited by PBN and BHT but not trolox. Representative traces of [Ca^2+^]*_i_*, as a function of time during a (**A**) 2 min change in external pH from 7.4 to 6.0 (black trace). Following a 20 min washout, a second acidosis insult (grey trace) induces an approximately 20% reduced elevation in [Ca^2+^]*_i_*. In separate experiments, following the control acidosis application (black traces), a 20 min incubation in (**B**) 100 μM PBN (red trace) or (**C**) 100 μM BHT (blue trace) produced decreases in the initial acidosis-induced increases in [Ca^2+^]*_i_*, greater than observed under control conditions; (**D**) 100 μM trolox failed to significantly decrease the initial increase in [Ca^2+^]*_i_* compared to control but did produce an elevated steady state level (green trace); (**E**) Summarizing experiments identical to (**A**–**D**), second responses recorded in the absence and presence of the three antioxidants were normalized to initial control responses and expressed as means ± S.E.M. Peak, initial [Ca^2+^]*_i_* increase relative to baseline; Steady State, [Ca^2+^]*_i_* measured at end of acidic episode, relative to baseline; Total Calcium, integration of [Ca^2+^]*_i_* increases from beginning of acidic event to end of record. One way ANOVA comparison was performed on ratios of test responses/control responses. * *p* < 0.5 compared to control, # *p* < 0.5 compared to trolox, † *p* < 0.5 compared to BHT. Number of cells measured; Control, *n* = 280; PBN, *n* = 107; BHT, *n* = 138; Trolox, *n* = 234.

Brief applications of acidosis have not been shown to produce elevations in ROS, but do activate ASIC1a channels and elevate [Ca^2+^]*_i_* in cortical neurons [1]. These increases in [Ca^2+^]*_i_* are due to activation of ASIC1a and downstream channels, including voltage-gated Ca^2+^ channels [1]. Thus, to determine if antioxidants could reduce elevations in [Ca^2+^]*_i_* produced by activation of ACIC1a, we examined changes in [Ca^2+^]*_i_* following brief exposure of cortical neurons to acidosis. Fura-2 loaded cultured cortical neurons incubated in PSS pH 7.4 were exposed for 2 min to PSS pH 6.0. Neurons responded with rapid, transient increases in [Ca^2+^]*_i_* (479 ± 32 nM), which decreased to baseline levels (30 ± 1 nM) within 1 min (Figure 2A). Incubation in 100 μM PBN or BHT for 20 min inhibited the initial acidosis induced peak increase by 36% and 40%, respectively (Figure 2B,C,E). However, trolox failed to significantly attenuate acidosis-evoked elevations in [Ca^2+^]*_i_* (Figure 2D,E). All three antioxidants produced elevated steady state levels at the end of the 2 min acidosis application, compared to control (PBN 20%, BHT 2% and trolox 8%) (Figure 2B–E). The discrepancy between the three antioxidants on [Ca^2+^]*_i_* increases caused by activation of ASIC1a suggests that neither a direct effect on ASIC1a or downstream Ca^2+^ channels, nor a decrease in ROS production alone, is responsible for the effects observed here or with ischemia alone.

### 2.3. PBN and BHT Inhibit the Initial Phase of the Biphasic Increase in [Ca^2+^]*_i_* Induced by Acidic Ischemia and Increase the Steady State Levels. PBN, BHT and Trolox Inhibit the Rebound Increase Observed Following Washout of the Acidic Ischemia Solution

**Figure 3.**
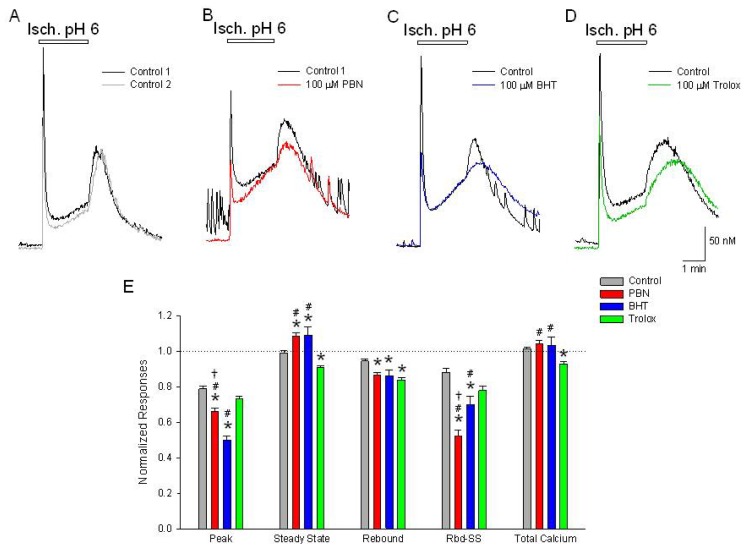
Initial [Ca^2+^]*_i_*, increases in response to acidic ischemia are inhibited by PBN and BHT. Representative traces of [Ca^2+^]*_i_*, as a function of time during a (**A**) 2 min application of glucose-free PSS pH 6.0 with 4 mM NaN_3_ (black trace). Following a 20 min washout, a second application of acidic ischemia induces an approximately equal [Ca^2+^]*_i_* response except for a 20% reduction of the initial peak elevation (grey trace). In separate experiments, following the control acidic ischemia application (black traces), cells were incubated for 20 min in (**B**) 100 μM PBN or (**C**) 100 μM BHT prior to a second acidic ischemia insult which produced decreases in the initial elevations in [Ca^2+^]*_i_* and in the rebound peaks following washout, but increased the steady state levels, measured immediately prior to washout, compared to control conditions (red and blue traces, respectively). Incubation in 100 μM trolox (**D**) failed to significantly decrease the initial increase in [Ca^2+^]*_i_* compared to control but did produce a decrease in the rebound peak (green trace); (**E**) Summarizing experiments identical to (**A**–**D**), second responses recorded in the absence and presence of the three antioxidants were normalized to initial control responses and expressed as means ± S.E.M. Peak, initial [Ca^2+^]*_i_* increase relative to baseline; Steady State, [Ca^2+^]*_i_* measured at end of acidic ischemia episode, relative to baseline; Rebound, [Ca^2+^]*_i_* increase observed after acidic ischemia event measured from baseline (pre-acidic ischemia); Rbd-SS, [Ca^2+^]*_i_* increase observed after acidic ischemia event measured from steady state level (end of acidic ischemia); Total Calcium, integration of [Ca^2+^]*_i_* increases from beginning of acidic ischemia event to end of record. One way ANOVA comparison was performed on ratios of test responses/control responses. * *p* < 0.5 compared to control, # *p* < 0.5 compared to trolox, † *p* < 0.5 compared to BHT. Number of cells measured; Control, *n* = 271; PBN, *n* = 107; BHT, *n* = 176; Trolox, *n* = 128.

During stroke, neurons are exposed to a multifactorial insult involving both ischemia and acidosis [15]. Our laboratory has shown that concurrent ischemia and acidosis synergistically potentiates [Ca^2+^]*_i_* overload, compared to ischemia or acidosis alone, which likely explains the increased neuronal death observed under these condition [2]. To determine how antioxidants affect these changes in [Ca^2+^]*_i_*, cortical neurons were exposed to an acidic ischemia solution of glucose-free PSS pH 6.0 with 4 mM NaN_3_ (Isch. pH 6). Neurons responded with rapid, transient increases in [Ca^2+^]*_i_* (304 ± 12 nM) followed by slow sustained increases (22.3 ± 1.1 nM/min) that continued for the duration of the exposure (Figure 3A). Upon washout of the acidic ischemia solution, the [Ca^2+^]*_i_* rebounded with increases (67 ± 3 nM) that peaked within 30 seconds before returning to baseline levels within 5–10 min (Figure 3A). Similar to responses to a second acidosis application, a second application of acidic ischemia, 20 min after the first insult, resulted in responses with a 20% reduction in initial peak increases (Figure 3A). Incubation in 100 μM PBN or BHT for 20 min inhibited the initial peak increases by 34% and 50% respectively, while elevating the steady state level, measured at the end of the acidic ischemia insult, by 9% (Figure 3B,C,E. Trolox, however, did not significantly reduce the initial peak, but did lower the steady state level by 9% (Figure 3D,E). All three antioxidants reduced the peak rebound [Ca^2+^]*_i_* elevation measured with respect to baseline (PBN—14%, BHT—14% and trolox—16%) (Figure 3E). The relative rebound peak, measured with respect to the steady state [Ca^2+^]*_i_* immediately prior to washout, was only significantly reduced by PBN, 48%, and BHT, 30% (Figure 3E). As with the individual ischemia or acidosis exposures, the antioxidants did not produce a consistent pattern of [Ca^2+^]*_i_* modulation following acidic ischemia.

### 2.4. PBN and BHT Potentiate the Rates of Increase in [Ca^2+^]*_i_*, during the Second Phase of the Response of Neurons to Acidic Ischemia

**Figure 4.**
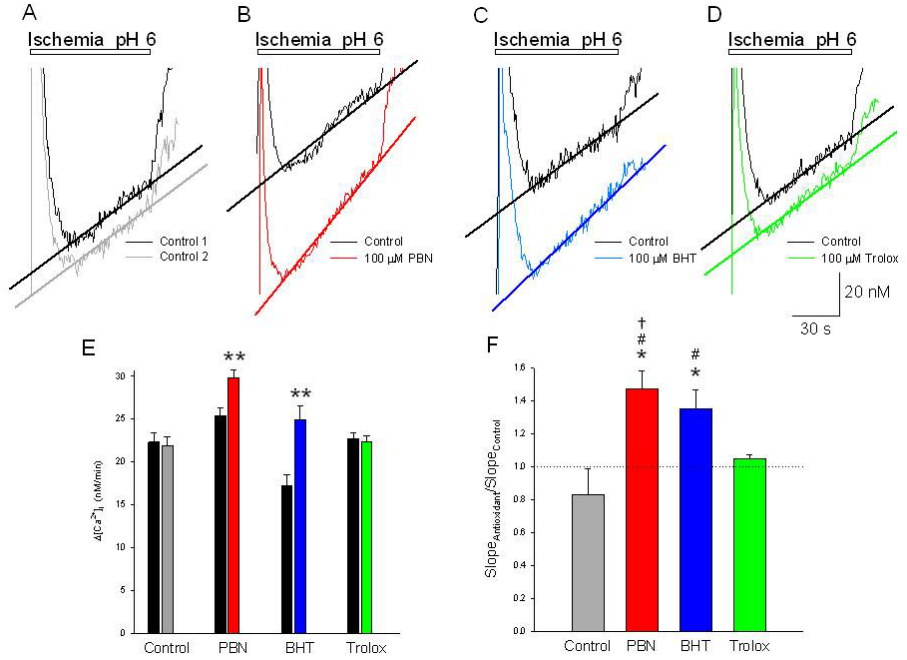
The rate of [Ca^2+^]*_i_*, increases in response to acidic ischemia are potentiated by PBN and BHT. Expanded, representative traces of the second phase of [Ca^2+^]*_i_*, increases as a function of time during a (**A**) 2 min application of glucose-free PSS pH 6.0 with 4 mM NaN_3_ (black trace). Following a 20 min washout, a second application of acidic ischemia induces an approximately equal rate of [Ca^2+^]*_i_* increase (grey trace); Straight lines are linear fits to the data. In separate experiments, following the control acidic ischemia application (black traces), cells were incubated for 20 min in (**B**) 100 μM PBN or (**C**) 100 μM BHT prior to a second acidic ischemia insult which produced rates of increase in [Ca^2+^]*_i_* greater than control values (red and blue traces, respectively). Incubation in 100 μM trolox (**D**) had no effect on the rate of increase in [Ca^2+^]*_i_* compared to control (green trace); (**E**) Summary of measurements of the slopes of [Ca^2+^]*_i_* increases from experiments identical to (**A**–**D**) expressed as means ± S.E.M.. Black bars represent slopes measured from initial control responses and color bars represent slopes from subsequent responses in the absence (gray) and presence of the antioxidants. ** *p* < 0.001, compared to control by student *t*-test; (**F**) Slopes measured from second responses to acidic ischemia recorded in the absence and presence of the three antioxidants were normalized to slopes measured from initial control responses and expressed as means ± S.E.M. One way ANOVA comparison was performed on ratios of test responses/control responses. * *p* < 0.5 compared to control, # *p* < 0.5 compared to trolox, † *p* < 0.5 compared to BHT. Number of cells measured; Control, *n* = 272; PBN, *n* = 106; BHT, *n* = 178; Trolox, *n* = 131.

After the initial rapid transient increase in [Ca^2+^]*_i_* associated with activation of ASIC channels, continued exposure to glucose-free PSS pH 6.0 with 4 mM NaN_3_ produces a linear increase in [Ca^2+^]*_i_*, 22.3 ± 1.1 nM/min, that persists for the duration of the exposure (Figure 4A). Such an increase would be consistent with a gradual accumulation of free radicals in the presence of ischemia. However, following incubation in 100 μM PBN or BHT, the rate of change of [Ca^2+^]*_i_* increases 50% and 40%, respectively (Figure 4B,C). In contrast, 100 μM trolox had no effect on this component of the rise in [Ca^2+^]*_i_*.

### 2.5. BHT Inhibits the Initial Increase in Inward Current Activated by Ischemia

**Figure 5.**
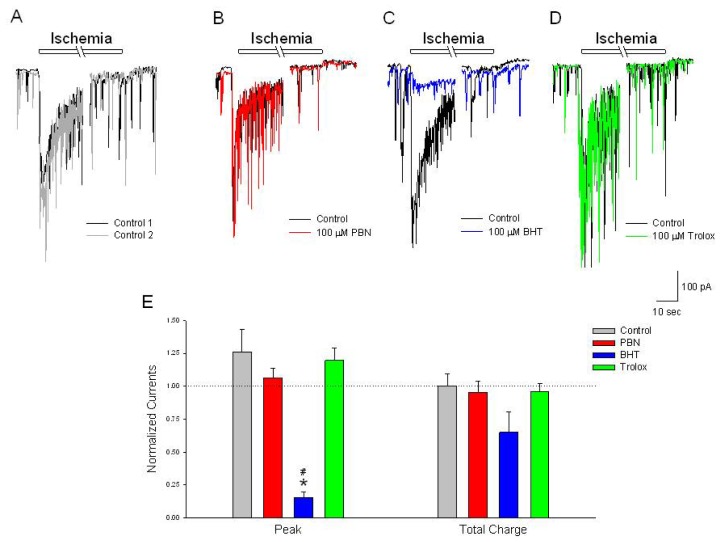
BHT inhibits the initial peak inward current activated by ischemia. (**A**–**D**) Representative traces of inward currents recorded from cultured rat cortical neurons voltage clamped at −70 mV in the perforated-patch whole-cell configuration. Control recordings (black and grey traces) demonstrate a rapid activating, slow inactivating current induced by a 2 min application of NaN_3_ in the absence of glucose. In separate experiments, following the control ischemic responses (black traces), a 20 min incubation in (**B**) 100 μM PBN (red trace) or (**D**) 100 μM trolox (green trace) had no effects on the ischemia activated current; (**C**) 100 μM BHT, however, inhibited the initial peak inward current (blue trace); (**E**) Summarizing experiments identical to (**A**–**D**), second responses recorded in the absence and presence of the three antioxidants were normalized to initial control responses and expressed as means ± S.E.M. Peak, initial inward current relative to baseline; Total Charge, integration of inward current from beginning of acidic ischemia event to end of record. One way ANOVA comparison was performed on ratios of test responses/control responses. * *p* < 0.5 compared to control, # *p* < 0.5 compared to trolox. Number of cells measured; Control, *n* = 6; PBN, *n* = 7; BHT, *n* = 6; Trolox, *n* = 5.

To further characterize responses to ischemia and acidosis in rat neurons, whole cell membrane currents were studied using patch clamp techniques in the perforated-patch whole cell configuration. Chemical ischemia activated inward currents in cells voltage clamped at −70 mV, characterized by a large initial transient (−589 ± 63 pA), which decayed to a sustained steady state current (−16 ± 11 pA) (Figure 5A). These currents were also observed upon a second ischemic even after a 20 min washout (−695 ± 64 and −14 ± 9 pA, respectively). Incubation in 100 μM PBN or trolox for 20 min had no effect on this current, while 100 μM BHT inhibited the initial peak current by 85% and increased the sustained inward current by 55 pA (Figure 5B–E).

### 2.6. None of the Antioxidants Significantly Inhibit the Initial Increase in Inward Current Activated by Acidosis

**Figure 6.**
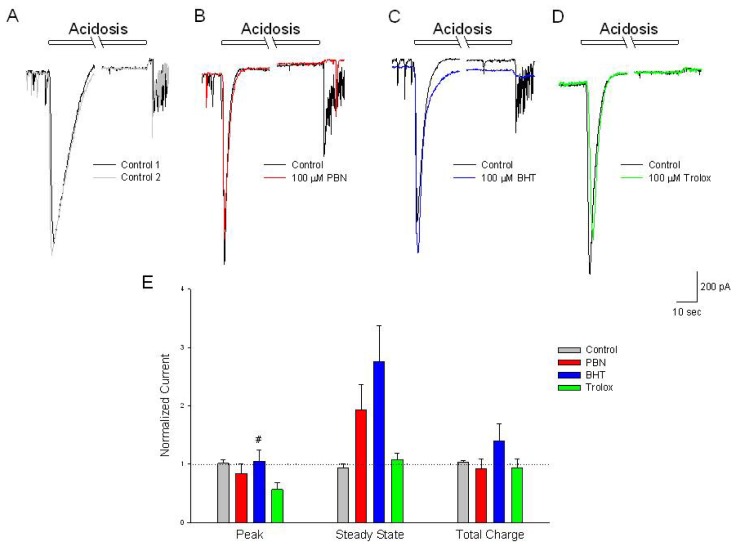
Antioxidants have no effect on acidosis-activated inward currents compared to control. (**A**–**D**) Representative traces of inward currents recorded from cultured rat cortical neurons voltage clamped at −70 mV in the perforated-patch whole-cell configuration. Control recordings (black and grey traces) demonstrate a rapid activating, inactivating current along with a sustained outward current, induced by a 2 min change in external pH from 7.4 to 6.0. In separate experiments, following the control acidosis responses (black traces); a 20 min incubation in (**B**) 100 μM PBN (red trace); (**C**) 100 μM BHT (blue trace) or (**D**) 100 μM trolox (green trace) had no effects on the acidosis activated currents; (**E**) Summarizing experiments identical to (**A**–**D**), second responses recorded in the absence and presence of the three antioxidants were normalized to initial control responses and expressed as means ± S.E.M. Peak, initial inward current relative to baseline; Steady State, current measured at end of acidic episode relative to baseline; Total Charge, integration of inward current from beginning of acidic ischemia event to end of record. One way ANOVA comparison was performed on ratios of test responses/control responses. # *p* < 0.5 compared to trolox. Number of cells measured; Control, *n* = 6; PBN, *n* = 5; BHT, *n* = 6; Trolox, *n* = 5.

Acidosis activates inward currents in cells voltage clamped at −70 mV, characterized by a large initial transient (−812 ± 192 pA) which rapidly inactivates, becoming an outward current (30 ± 12 pA) which are reproducible after a 20 min washout (−897 ± 64 and 45 ± 21 pA, respectively) (Figure 6A). Incubation in 100 μM PBN, BHT or trolox for 20 min had no statistically significant effects on this current (Figure 6B–E). Interestingly, peak currents measured in the presence of BHT were significantly different from currents measured in the presence of trolox (Figure 6E).

### 2.7. BHT Potentiates the Steady State Inward Current Activated by Acidic Ischemia

**Figure 7.**
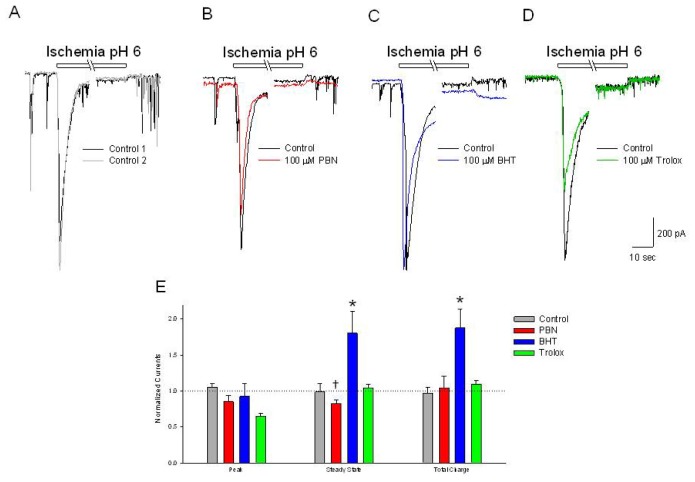
Trolox decreases initial transient acidic ischemia-activated inward currents while BHT increases the sustained activated currents. (**A**–**D**) Representative traces of inward currents recorded from cultured rat cortical neurons voltage clamped at −70 mV in the perforated-patch whole-cell configuration. Control recordings (black and grey traces) demonstrate a rapid activating, inactivating current induced by 2 min acidic ischemia. In separate experiments, following the control acidic ischemia responses (black traces); a 20 min incubation in (**B**) 100 μM PBN (red trace) had not effect; (**C**) 100 μM BHT (blue trace) increased the steady state inward current and (**D**) 100 μM trolox (green trace) decreased the initial transient inward current; (**E**) Summarizing experiments identical to (**A**–**D**), second responses recorded in the absence and presence of the three antioxidants were normalized to initial control responses and expressed as means ± S.E.M. Peak, initial inward current relative to baseline; Steady State, current measured at end of acidic episode relative to baseline; Total Charge, integration of inward current from beginning of acidic ischemia event to end of record. One way ANOVA comparison was performed on ratios of test responses/control responses. * *p* < 0.5 compared to control, † *p* < 0.5 compared to BHT. Number of cells measured; Control, *n* = 6; PBN, *n* = 6; BHT, *n* = 5; Trolox, *n* = 4.

Acidic ischemia activates inward currents in cells voltage clamped at −70 mV, characterized by a large initial transient (−1201 ± 179 pA), which rapidly inactivates to baseline (−28 ± 31 pA) (Figure 7A). Activation of this current was reproducible following a 20 min washout (−1366 ± 186 and −45 ± 54 pA, respectively). Incubation in 100 μM PBN or 100 μM BHT for 20 min had no effect on the initial transient current (Figure 7B–E). BHT, however, potentiated the inward steady state current by 80% (Figure 7E). Incubation in 100 μM trolox produced a 35% reduction in the initial inward current, which was not statistically different from control when compared to all the treatments using a one-way ANOVA, but was statistically different from control when compared with a student *t*-test (*p* < 0.001) (Figure 7E).

### 2.8. Discussion

Ischemia, acidosis and their combination lead to neuronal death following stroke. An initial event caused by ischemia and acidosis is disruption of ionic homeostasis, which in turn produces membrane depolarization and current activation. Under these conditions, mitochondrial stress leads to the formation of free radicals. The inconsistent results obtained using three different antioxidants suggest that free radical formation is downstream from the elevations in [Ca^2+^]*_i_* and current activation observed following ischemia and acidosis. BHT and trolox, α-tocopherol (Vitamin E) analogues, had significantly different effects, while BHT and PBN had similar effects on [Ca^2+^]*_i_* responses. The only consistent result obtained was that all three antioxidants attenuated the magnitude of the rebound [Ca^2+^]*_i_* increase observed upon washout of the acidic ischemia solution. There are reports in the literature that postulate the sodium-calcium exchanger (NCX) is inhibited during hypoxia and requires ROS for reactivation upon reperfusion [16]. Acidosis activates the ASIC channel, a member of the ENaC family, producing a Na^+^ influx, facilitating operation of NCX in reverse mode. The observed increase in [Ca^2+^]*_i_* following washout would be consistent with relief of inhibition of the exchanger. According to Eigel *et al.* [16], the magnitude of this relief is dependent on the concentration of ROS and thus sensitive to the presence of antioxidants. PBN had no effects on activated currents. Trolox inhibited initial peak currents activated by acidosis and acidic ischemia. Under conditions used in these experiments, this initial transient inward current is predominately a sodium current through the ASIC channel. In contrast, BHT inhibited the slower activating initial ischemia-induced currents, which are likely glutamate activated NMDA currents. These results suggest that during these acute insults PBN, BHT and trolox are not effecting free radical levels as much as they are having non-specific effects on the channels and exchangers responsible for the disruption of calcium homeostasis and current activation observed during ischemia, acidosis and ischemic acidosis.

## 3. Experimental Section

### 3.1. Primary Rat Cortical Neuron Preparation

Primary cortical neurons from embryonic (E18) rats were isolated and cultured, as previously described [14]. Excised brains were digested with 0.25% trypsin, cells suspended in DMEM supplemented with fetal bovine serum (10%, heat inactivated), penicillin (100 IU/mL), streptomycin (100 μg/mL) and amphotericin B1 (0.25 μg/mL) and plated on poly-l-lysine coated coverslips. Following 24 h incubation, the DMEM solution was replaced with Neurobasal media supplemented with B-27 and 0.5 mM l-glutamine. Both solutions contained 25 mM glucose and the Neurobasal media contained 11 mM HEPES. All procedures were done in accordance with the regulations of the University of South Florida Institutional Animal Care and Use Committee (Tampa, FL, USA). Cells were used after 10–21 days in culture.

### 3.2. Calcium Imaging Measurements

Changes in intracellular Ca^2+^ concentrations, [Ca^2+^]*_i_*, were examined in isolated cortical neurons using fluorescent imaging techniques and the Ca^2+^ sensitive dye, fura-2. Experimental protocols and calibration procedures were previously described [14]. Cells were loaded with the membrane permeable ester form of fura-2, fura-2 acetoxymethyl ester (fura-2 AM) [17], which becomes hydrolyzed by endogenous esterase activity into a membrane impermeable salt. Cells plated on coverslips were incubated for 1 h at room temperature in Neurobasal media with 4 μM fura-2 AM and 0.4% dimethyl sulfoxide. The coverslips were washed in a physiological saline solution (PSS) (fura-2 AM free) prior to experiments being performed.

### 3.3. Electrophysiological Measurements

Whole cell membrane currents were recorded using protocols previously described [1]. Briefly, neurons plated on poly-l-lysine coated glass coverslips were transferred to a recording chamber and membrane currents were amplified, filtered at 240 Hz, digitized at 50 Hz, and acquired using Clampex 9 (Molecular Devices, Sunnyvale, CA, USA), an Axon 200 amplifier and a Digidata 1322A digitizer (Molecular Devices, Sunnyvale, CA, USA). Electrical access was achieved using amphotericin B to perforate on-cell patches, preserving the intracellular integrity of the neurons [18]. An amphotericin B stock solution (60 mg/mL in DMSO) was made fresh daily and diluted to 240 μg/mL (0.4% DMSO) in control pipette solution, immediately prior to seal formation. Patch electrodes were pulled from thin-walled borosilicate glass (World Precision Instruments Inc., Sarasota, FL, USA) using a Sutter Instruments P-87 pipette puller (Novato, CA, USA) and had resistances of 1.0–1.5 MΩ. Access resistances (*R*_s_) were monitored throughout experiments for stable values ≤30 MΩ and were always compensated at 40% (lag, 10 μs).

### 3.4. Solutions and Reagents

The control bath solution for all experiments was PSS. For experiments examining only ischemia, the PSS contained (mM): 140 NaCl, 3 KCl, 2.5 CaCl_2_, 1.2 MgCl_2_, 7.7 glucose, and 10 HEPES, pH to 7.2 with NaOH. Chemical ischemia was induced using this solution in the absence of glucose and with 4 mM NaN_3_. For experiments examining acidosis and acidic ischemia the PSS contained (mM) 140 NaCl, 5.4 KCl, 1.3 CaCl_2_, 1.0 MgCl_2_, 20 glucose, and 25 HEPES (pH to 7.4 with NaOH). Acidosis was induced using this solution at pH 6.0. Acidic ischemia was induced using this solution in the absence of glucose with 4 mM NaN_3_, pH 6.0. Solutions were applied using a rapid application system identical to that previously described [19]. Individual cells were exposed to 2 ischemic, acidic or acidic ischemia insults with 20 min washes between episodes to minimize rundown of responses [1,14]. Antioxidants were applied for 20 min prior to and during ischemia/acidosis insults. The control pipette solution consisted of (in mM): 75 K_2_SO_4_, 55 KCl, 5 MgSO_4_, and 10 HEPES (titrated to pH 7.2 with *N*-methyl-d-glucamine). All chemicals used in this investigation were of analytical grade. The following drugs were used: amphotericin B, dimethyl sulfoxide (DMSO), trolox, butylated hydroxytoluene (BHT), *N*-tert-butyl-α-phenylnitrone (PBN), sodium-azide (Sigma-Aldrich, St. Louis, MO, USA); and fura-2 AM (Molecular Probes, Eugene, OR, USA).

### 3.5. Data Analysis

Imaging data files were collected with SlideBook 4.02 (Intelligent Imaging Innovations, Inc., Denver, CO, USA). Intensities of individual fluorescent cells were measured as functions of time using SlideBook (Intelligent Imaging Innovations, Inc., Denver, CO, USA), converted to [Ca^2+^]*_i_*, values and exported using SigmaPlot 11 (Systat, San Jose, CA, USA). Analyses of [Ca^2+^]*_i_* responses and electrophysiological recordings and were performed using Clampfit 9 (Molecular Devices, Sunnyvale, CA, USA) and consisted of measuring initial and rebound peak values, calculating average baseline and steady state values, performing linear fits to determine slopes and integrating responses to calculate total calcium and charge. Statistical analysis was conducted using SigmaPlot 11 (Systat, San Jose, CA, USA). Statistical differences were determined using paired and unpaired *t*-tests for within group and between group experiments, respectively, and were considered significant if *p* < 0.05. For multiple group comparisons a 1-way ANOVA, with or without repeat measures, were used, as appropriate. When significant differences were determined with an ANOVA, *post-hoc* analysis was conducted using a Tukey Test to determine differences between individual groups.

## 4. Conclusions

The results presented here are not consistent with PBN, BHT and trolox effecting [Ca^2+^]*_i_* and membrane current responses to acute ischemia and acidosis by eliminating free radicals. While ER and mitochondrial stress do lead to production of free radicals, the inconsistent results obtained using these three antioxidants, particularly the two α-tocopherol (vitamin E) analogues, suggests non-specific effects are responsible for inhibition and potentiation of [Ca^2+^]*_i_* and whole-cell current changes. The disruption of ionic homeostasis in response to ischemia and acidosis observed involves a myriad of ion channels, exchangers and ATPases, all possible targets for modulation by PBN, BHT and trolox.
